# Cardiorespiratory Optimal Point: A Submaximal Exercise Variable to Assess Panic Disorder Patients

**DOI:** 10.1371/journal.pone.0104932

**Published:** 2014-08-26

**Authors:** Plínio Santos Ramos, Aline Sardinha, Antonio Egidio Nardi, Claudio Gil Soares de Araújo

**Affiliations:** 1 Exercise Medicine Clinic – CLINIMEX, Rio de Janeiro, Rio de Janeiro, Brazil; 2 Maternity Hospital Therezinha de Jesus, Faculty of Medical and Health Sciences – SUPREMA, Juiz de Fora, Minas Gerais, Brazil; 3 Panic and Respiration Laboratory, Institute of Psychiatry, Federal University of Rio de Janeiro, National Institute for Translational Medicine (INCT-TM/CNPq), Rio de Janeiro, Rio de Janeiro, Brazil; 4 Heart Institute Edson Saad, Federal University of Rio de Janeiro, Rio de Janeiro, Rio de Janeiro, Brazil; Emory University, United States of America

## Abstract

Panic disorder (PD) patients often report respiratory symptoms and tend to perform poorly during maximal cardiopulmonary exercise testing (CPX), at least partially, due to phobic anxiety. Thus, we hypothesized that a submaximal exercise variable, minimum VE/VO_2_ - hereafter named cardiorespiratory optimal point (COP) -, may be useful in their clinical assessment. Data from 2,338 subjects were retrospectively analyzed and 52 (2.2%) patients diagnosed with PD (PDG) (70% women; aged 48±13 years). PD patients were compared with a healthy control group (CG) precisely matched to number of cases, age and gender profiles. PDG was further divided into two subgroups, based on having achieved a maximal or a submaximal CPX (unwilling to continue until exhaustion). We compared COP, VO_2_ max, maximum heart rate (HR max) between PDG and CG, and also COP between maximal and submaximal PD subgroups. COP was similar between PDG and CG (21.9±0.5 vs. 23.4±0.6; p = 0.07), as well as, for PD subgroups of maximal and submaximal CPX (22.0±0.5 vs. 21.6±1.3; p = 0.746). Additionally, PD patients completing a maximal CPX obtained VO_2_ max (mL.kg−1.min−1) (32.9±1.57 vs 29.6±1.48; p = 0.145) and HR max (bpm) similar to controls (173±2.0 vs 168±2.7; p = 0.178). No adverse complications occurred during CPX. Although clinically safe, it is sometimes difficult to obtain a true maximal CPX in PD patients. Normalcy of cardiorespiratory interaction at submaximal effort as assessed by COP may contribute to reassure both patients and physicians that there is no physiological substrate for exercise-related respiratory symptoms often reported by PD patients.

## Introduction

Patients reporting exercise-induced shortness of breath and/or palpitations are often anxious [1]. They could be a real diagnostic challenge since it is quite often difficult to distinguish between a real clinical abnormal respiratory or circulatory condition from one commonly seen during panic attacks (PA) [2], [3]. Despite a very high lifetime prevalence of about 28% [4] for PA in adult subjects, It is worthwhile to note there are very limited amount of data about exercise and panic and, more specifically, about what role, if any, could cardiopulmonary exercise testing play in the clinical assessment of these patients.

Panic disorder (PD) is characterized by recurrent and unexpected anxiety or PA, without apparent cause, PA are characterized by the sudden appearance of four or more autonomic and respiratory symptoms, reaching peak in approximately 10 minutes and then disappearing [5]. Some evidence suggests that these patients may have subclinical respiratory and other abnormalities [3] related to body homeostasis during rest [2], as, for example, hyperventilation syndrome [6], with increased sensitivity to carbon dioxide (CO_2_) [7], [8]. This increased CO_2_ sensitivity led to the most well accepted current hypothesis to explain the occurrence of PA, the theory of false suffocation alarm, that is related to abnormal control mechanisms of respiration in these patients [9].

It is well-established that expired gas data obtained during cardiopulmonary exercise testing (CPX) can be used in the diagnosis and prognosis of numerous pathologic conditions [10], [11]. however, there are very limited data on CPX in PD patients [12]. In fact, preliminary evidence suggests that aerobic fitness may be reduced in this clinical condition [13], [14], [15]. Additionally, performance of maximal CPX could be particularly hampered by phobic anxiety, with autonomic manifestations naturally triggered by exercise, similar to those present in a PA [16]. Thus, due to the anticipatory anxiety related to a PA while performing a CPX, it is quite common that the patient will be unwilling to achieve the exhaustion that characterizes a maximal CPX [13], [17].

In this study, we have used a recently proposed submaximal exercise variable, the cardiorespiratory optimal point (COP) [18], which represents the minimal ventilation (VE)/oxygen uptake (VO_2_) value and reflects the best circulatory-respiratory interaction, to assess exercise ventilatory response in PD patients. So, in order to test the hypothesis that the exercise-related respiratory symptoms in PD patients are not due to a true abnormal cardiorespiratory interaction, we compared COP during CPX in PD patients and age and gender-matched healthy subjects. If our hypothesis is correct this will reassure both patients and physicians about the absence of a true physiological substrate for these frequent patients' complains.

## Materials and Methods

### Ethics statement

Both consent form and all study procedures and retrospective data analyses were previously approved by the Faculty of Medical and Health Sciences, SUPREMA institutional research ethics committee under number (protocol number 0166.0.000.399-11) in accordance with the Declaration of Helsinki of the World Medical Association Ethics Code. All subjects participated in the present study voluntarily, receiving no financial incentive. Participants were informed that they could withdraw at any time. Each subject (legal guardian if minor) read and signed a specific informed consent form.

All data are only available upon request to the senior author (legal guardian of the data). This option follows the local laws and ethical regulations that apply to confidentiality of clinical/medical data.

### Sample

Among 2,338 subjects evaluated in our Exercise Medicine Clinic between January, 2006 and September, 2012, those who were previously diagnosed with PD by their doctors, according to the DSM-IV [5], and who met the following inclusion criteria: 1) absence of known cardiopulmonary diseases; 2) not under regular use of relevant medications; 3) body mass index <30 kg/m^2^; 4) normal resting spirometry (FEV1/FVC>70%) [19]; 5) absence of clinical or electrocardiographic changes that characterize myocardial ischemia during CPX and 6) absence of locomotor limitations that can hinder the implementation of lower-limb cycling CPX. Among the 83 patients identified with PD (3.6% of total), 31 were excluded, nine for having performed treadmill CPX, 13 due to obesity, three due to a history and/or signs of coronary artery disease to CPX and six due to abnormal resting spirometry. To the remaining 52 patients, age was 48±13 years (mean±standard deviation), 36 (70%) were women, 36 (70%) had current clinical PD presentation and 16 (30%) had a history and a previous diagnosis of PD (without recurrence of PA in the last six months).

### Control Group

A control group (CG) precisely matched with the same number of cases and the same age and sex profile as the PD patients was selected from a database of 624 healthy individuals evaluated during the same period that also attended all the inclusion criteria. Selection was performed from numbers assigned to each healthy subject belonging to the database by using the site www.randomizer.org.

### Characterization of Subgroups

Two groups were so defined: healthy individuals (CG) and PD patients (PDG). For some further analyses, PDG was divided in two subgroups, MAX (maximal) and SUBMAX (submaximal) based on whether patients effectively concluded a truly maximal CPX.

### Evaluation Protocol

Evaluation consisted of medical history, including specific questions about diagnosis, occurrence of PA events and treatment of PD and physical examination. Additionally, all medications in regular use were recorded. Anthropometric data was collected and resting spirometry and 12-lead electrocardiogram obtained. Subjects volunteered for the evaluation, typically performed under request of their attending physicians before to start an exercise program.

### Resting Spirometry

At least three flow-volume curve maneuvers were performed using a pneumotachograph (SP-1 Spirometer, Schiller, Switzerland), periodically calibrated according to the standard recommendations [20]. Maximal voluntary ventilation was predicted by considering forced expiratory volume in one second (FEV1)×40 [21].

### Maximal Cardiopulmonary Exercise Testing (CPX)

All subjects underwent a cycling maximal CPX (Cateye EC-1600, Cateye, Japan or Inbrasport CG-04, Inbrasport, Brazil), following an individualized ramp protocol aimed to obtain exhaustion between eight and 12 minutes [22]. The CPX's maximal nature was subjectively defined by the well-trained physician who supervised the procedure and supported by physiological indicators, such as the occurrence of anaerobic threshold (AT) [23], a U pattern of the ventilatory equivalent curves - and by – a score of 10 on the 0–10 Borg scale. The CPXs of patients who did not simultaneously achieve all of these criteria were considered submaximal [24], [25]. Throughout CPX, electrocardiogram, heart rate, blood pressure and arterial oxygen saturation were also obtained.

### Expired Gases Analyses

During CPX, expired gases were collected using a pneumotachograph Prevent (MedGraphics, USA) coupled to a mouthpiece, with concomitant nasal occlusion. A metabolic analyzer VO2000 (MedGraphics, United States) enabled the quantification of VE and partial fractions of oxygen and carbon dioxide, expressed every 10 s and later recalculated and expressed as mean values for each minute of CPX. These devices were regularly calibrated by a 2-L syringe and gases of known concentrations.

### Cardiorespiratory Optimal Point (COP)

COP, a dimensionless variable, is defined as the minimal ventilation that is used for the consumption of one liter of oxygen, i.e, the identification of smallest value of the minute-by-minute ratio of VE and VO_2_ during CPX and reflects the best possible cardiorespiratory interaction during exercise. The identification of COP is quite simple, objective, and independent from the evaluator's judgment. COP reference values for a wide age range among men and women are already available favoring its interpretation [18] and its measurement has been shown to be reliable in two separate CPX performed in the same subjects [26].

### Statistical Analyses

Initially, normality (Kolmogorov-Smirnov) and homoscedasticity of the data distribution were tested. For descriptive purposes, central tendency and data variability were expressed as mean±standard deviation (minimal and maximal), and for statistical inference, as mean±standard error of the mean. Groups' comparisons were carried out by Student's t-test, except for between COP of the MAX and SUBMAX comparisons that due to the non-parametric distribution was carried out by Mann-Whitney test. Prism software version 6 (GraphPad, USA) was used for statistical analysis, considering a probability level of 5%.

## Results

The most relevant demographic characteristics and the main results of resting spirometry and CPX, as a function of group/subgroup, are shown in Table 1.

**Table 1:** Characteristics and main results for spirometry and CPET in groups and subgroups.

Variables	Control		Panic Disorder	
Characteristics	All(n = 52)	All (n = 52)	Maximal (n = 41)	Submaximal(n = 11)
Sex (Female/Male)	31/21	31/21	20/21	11/0
Age (years)	47.7±13.2	48.5±13.4	47.5±13.6	52.5±12.0
	(19–75)	(17–78)	(17–74)	(34–78)
BMI (kg/m^2^)	24.2±2.7	24.3±3.1	24.1±3.1	25.1-3.1
	(18.5–29.5)	(19.2–30.0)	(19.2–30.0)	(19.8–29.2)
HR at rest (bpm)	66.2±12.8	66.8±11.0	66.8±11.3	66.8±10.1
	(48–96)	(43–96)	(43–96)	(50–88)
**Spirometry**				
FEV1/FVC (%)	82.4±5.1	81.5±5.2	82.2±5.3	79.0±3.7
	(73.6–93.3)	(70.0–95.5)	(70.0–.5)	(70.1–83.2)
**CPET**				
Total Time (min)	10.6±2.3	9.5±2.4	10.3±1.8	6.7±1.8
	(6–15)	(3.4–14.0)	(7.0–14.0)	(3.4–9.5)
Maximum HR (bpm)	172.6±14.8	164.7±20.0	168.0±17.3	149.0±22.5
	(143–200)	(111–197)	(120–197)	(111–180)
Maximum predict VO_2_ (%)	104.4±32.3	87.6±27.4	94.0±27.1	63.9±11.3
	(57.1–207.6)	(43.4–176.1)	(49.9–176.1)	(43.4–87.6)
Maximum VO_2_ (mL.(kg.min)−1)	32.9±11.3	27.2±9.8	29.6±9.4	18.1±3.5
	(14.3–58.5)	(12.3–63.0)	(12.5–63.0)	(12.3–22.6)
VO2 AT (mL.(kg.min)−1)	23.0±8.6	19.2±6.6	20.5±6.7	14.5±2.9
	(9.1–45.5)	(5.4–48.0)	(5.4–48.0)	(7.5–18.0)
Maximum VE (L.min^−1^)	81.9±31.7	61.1±27.3	68.1±25.7	35.0±14.4
	(34.9–160.2)	(21.1–139.0)	(23–139)	(21.1–61.4)
Maximal voluntary ventilation (VEF1×40) (L.min^−1^)	117.6±30.2	109.1±27.1	115.6±25.0	84.7±20.1
	(62.4–184.4)	(48.4–168.8)	(60.0–168.8)	(48.8–114.4)
COP (VE/VO_2_ min)	23.4±4.7	21.6±3.4	22.0±3.2	21.6±4.3
	(16.0–34.8)	(14.6–30.0)	(15.8–30.0)	(14.6–29.8)

BMI: Body mass index; HR: Heart Rate; FEV1: forced expiratory volume in 1 second; FVC: forced vital capacity; VO2: oxygen uptake; AT: Anaerobic Threshold; COP: cardiorespiratory optimal point.

**Figure 1:**
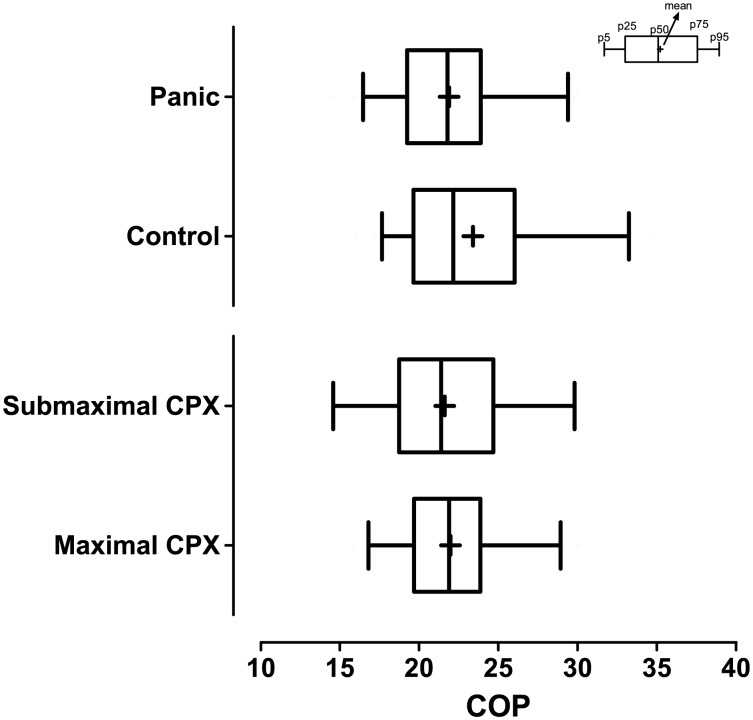
Comparison of COP for the control and PD groups and for the subgroups MAX and SUBMAX of patients with PD. * p<0.05.

When evaluating COP results, no differences between PD patients and control subjects (21.9±0.47 vs. 23.4±0.65; p = 0.067) nor between PDG SUBMAX and MAX CPX subgroups (22.0±0.50 vs. 21.6±1.31; p = 0.736) were found (Figure 1). In contrast, VO_2_ max (mL.(kg.min)-1) of PDG was lower than in CG (27.2±1.35 vs. 32.9±1.57; p<0.001) and HR max (bpm) were higher for CG than for PDG (173±2.0 vs. 164±2.8; p = 0.013) (Table 1).

While comparing those who had performed a maximal CPX, i.e., CG and PDG MAX subgroup, there were no differences in VO_2_ max (32.9±1.57 vs. 29.6±1.48 mL.kg^−1.^min^−1^; p = 0.135) or in HR max (173±2.0 vs. 168±2.7 bpm; p = 0.168).

**Figure 2:**
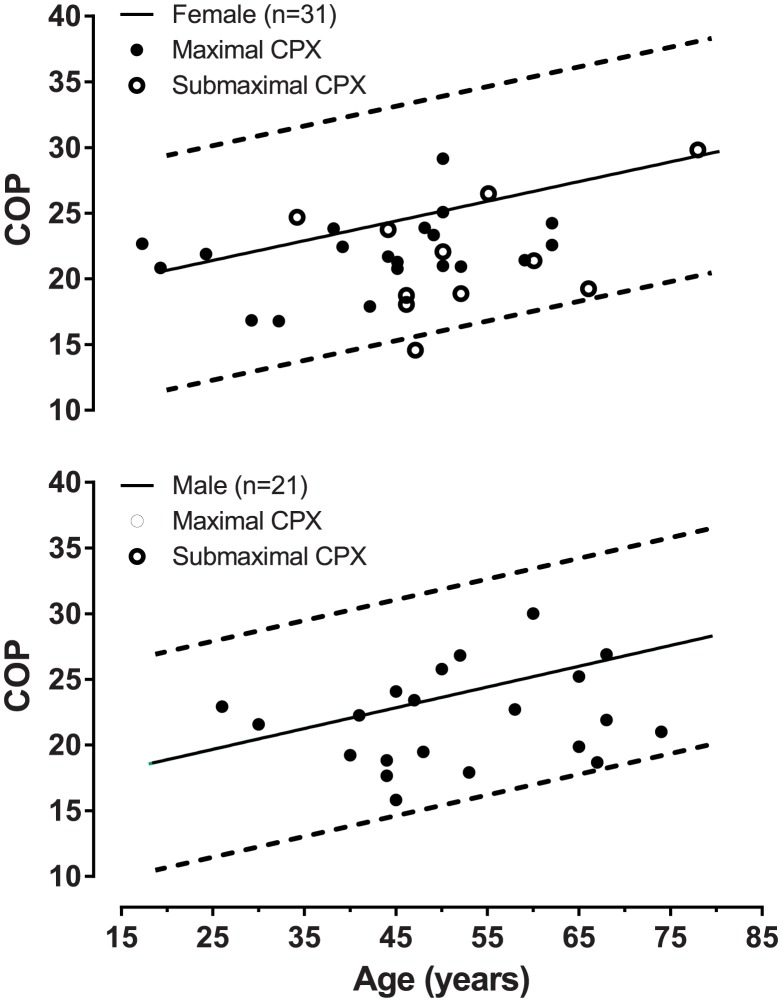
COP results for PD patients and maximal and submaximal CPET compared with the age and gender-predicted values.

Regarding COP results, except for one case (normality threshold value) all other 51 PD patients (98% of the total) had results that felt within the boundaries of two standard errors from the expected COP age-and-sex reference value [18]. (Figure 2).

It is noteworthy that none of PD patients had abnormal clinical, hemodynamic - blood pressure and heart rate - or respiratory responses to incremental exercise. Objectively, none of them achieved ventilatory limitation during CPX, as observed by VE maximal final values that were substantially below FEV1×40 [21]. (Table 1). Although minor ECG changes were eventually detected, such as isolated J point depression accompanied by rapid up slopping pattern of ST segment, it should be emphasized that there were no complaints of dyspnea or typical stress-induced precordial pain during or immediately following CPX by any of PD patients.

## Discussion

Considering the initial sample of 2,338 adults, the prevalence of 3.6% of PD, tends to be slightly higher than reported by others [27]. This is consistent with the fact that our laboratory is a reference center for referral of such patients. The present study has several relevant findings. First, it has a sample size considerably larger than other previous studies [13], [14], [28], [29] in which the exercise response of PD patients was assessed. Additionally, the PD patients' results could be compared with those obtained in a set of healthy subjects with identical age and sex distribution, drawn randomly from a large database of the same laboratory.

Consistent with few existing studies of maximal exercise testing in PD patients [13], [15], [30], our data confirm that CPX is clinically safe in these patients and that the induction of PA seems to be very rare (no cases in the present study), occurring in less than 0.5% of cases, when all reported cases in the literature are combined [13], [30]. Several studies have investigated respiratory symptoms in PD patients [2], [31], [32], [33] and found that, during PA, respiratory complaints are common, such as dyspnea and tachypnea. Some specific respiratory tests aimed at inducing PA have been used, demonstrating that respiratory maneuvers, such as hyperventilation [34], carbon dioxide inhalation [35] and voluntary apnea [36], may increase anxiety in PD patients and thereby trigger a PA. In this regard, several studies have contributed to better understand the relationship between respiration and PA. In a recent review [31], it has been suggested that during hyperventilation, there are hypocapnia and vasoconstriction, with decreased cerebral blood flow, which may induces a PA. On the other hand, stimulation of chemoreceptors resulting from hypercapnia caused by CO_2_ inhalation, may cause a hyperpnea that activates an abnormally sensitive fear network in the brainstem, and then also induce PA [31].

Exercise triggers physiological responses aimed to preserve homeostasis. In the respiratory system, there is increased VE mediated by a combination of hypercapnia, hypoxia, metabolic acidosis and afferent stimulation of joints and muscles [37], [38], several of them may be directly involved in PA. Thus, it is possible that clinical characteristics of PD patients hinder the achievement of maximal CPX variables. In this sense, other studies subjecting PD patients to exercise testing showed lower HR max and aerobic fitness, and that this occurs, at least in part, by the difficulty in obtaining a truly maximal test [14].

PD patients commonly exhibit greater sensitivity to autonomic symptoms, which are potentially responsible for the phobic avoidance of situations that potentially trigger PA, such as intense exercise [29], [39]. Since pre-exercise anxiety can lead to PAs, and some patients may require exercise discontinuation before reaching maximal effort, it could be difficult to determine the aerobic fitness of a PD patient. This issue can be further complicated during CPX when expired gases are collected. In fact, it has been previously reported [40] that breathing using a mouthpiece with occluded nose accompanied by the perception of increased HR can contribute to symptoms of discomfort, dyspnea, asphyxia and suffocation in PD patients. In the present study, approximately 20% of patients were unwilling to complete a truly maximal CPX, making submaximal cardiorespiratory data even more relevant. As previously stated, premature CPX interruption tends to do not affect COP determination, since COP occured at about 44% of VO_2_ max and so, well before anaerobic threshold and maximal effort [18]. This is further corroborated by the fact that PDG and CG did not have different COP results, and there was no difference in COP when comparing between subgroups of PD patients (Figure 1).

When investigating COP behavior of PD patients (Figure 2) by using a regression equation [18], our results show that COP was about always (except in a single case), within the boundaries of estimate of error for healthy subjects. Therefore, it was observed that, from the point of view of cardiorespiratory integration, reflecting, at least partially, the quality of ventilation-perfusion ratio during exercise, PD patients exhibit no limitations and behave similarly to healthy subjects. Our data are in agreement with Caldirola et al. [13] who also observed that despite a high level of anxiety of PD patients at rest and a lower VO_2_ followed by a higher HR during the exercise, treadmill ventilatory responses up to 65% of predicted HR max was similar between PD patients and healthy subjects.

In contrast, as already noted by other authors [13], [14], [28], VO_2_ max tends to be lower in PD patients than in age and sex-matched healthy subjects. Nevertheless, when healthy subjects are compared with PD patients' subgroup who achieved maximal CPX, these differences disappeared. This lower aerobic fitness presented in many PD patients may be associated with the avoidance of potentially anxiogenic activities, leading to a reduction of exposure to exercise and outside activities that could somehow improve their aerobic fitness [13]. Nevertheless, it should be noted that regular exercise improves this condition, leading to levels similar to that of healthy subjects [14].

Another clinically relevant point is that in PDG SUBMAX, all patients were women. There are robust evidence of true sex differences in regards to the vulnerability and the psychopathology of panic. [41], [42], [43]. Few hypotheses have been advocated in recent decades to explain the etiology of these sex-related differences in the presentation of PD, including the progesterone receptor polymorphism [41], a lesser sensitivity of beta-adrenergic receptors [43] and distinct processes of fear conditioning [42]. In clinical terms, this greater female sensitivity to anxiety makes them to became highly responsive to the perception of autonomic symptoms as it occurs during a PA. This increased sensitivity also potentiates the panicogenic effects [16] and it has been previously associated with exercise avoidance in PD patients [13], [39]. It is possible that this has contributed to explain why our female subjects prematurely stopped the CPX.

It should also be pointed that the present study has some limitations, such as the continued regular use of psychotropic drugs by PD patients. On the other hand, maintenance of the prescribed medication tends to increase the external validity of our results. Another potential limitation is the lack of a more detailed differentiation of the clinical condition of PD, as, for example, the classification of the PD patients in respiratory and non-respiratory subtypes, Nevertheless, a recent study [44] has found no significant differences in VO_2_ max according to the subtype.

In conclusion, our study showed that cardiorespiratory interaction as assessed by COP – a submaximal variable obtained during CPX – was found to be within age- and sex-defined normal limits in PD patients, even of those unable to complete a truly maximal CPX. Additionally, it was confirmed that CPX is clinically safe, very unlikely to induce PA and is indicated for the assessment of cardiorespiratory symptoms of PD patients, even when a maximal CPX is not reached, being able to provide clinically relevant information and reassuring health professionals about the absence of a true physiological substrate for these often reported symptoms.
